# Acute Bacterial Suppurative Thyroiditis following Fine Needle Aspiration: A Case Report and Review of the Literature

**DOI:** 10.1155/2020/7104806

**Published:** 2020-08-25

**Authors:** Zaw Win Htet, E. Shyong Tai, Samantha Peiling Yang

**Affiliations:** ^1^Division of Endocrinology, Department of Medicine, National University Hospital, Level 10, NUHS Tower Block, 1E Kent Ridge Road, Singapore 119228; ^2^Division of Endocrinology, Department of Medicine, Yong Loo Lin School of Medicine, National University of Singapore, Level 10, NUHS Tower Block, 1E Kent Ridge Road, Singapore 119228

## Abstract

**Background:**

Fine needle aspiration (FNA) of thyroid nodules is a simple, reliable, and inexpensive procedure performed on suspicious thyroid nodules found in thyroid ultrasound (US). Acute bacterial suppurative thyroiditis is an uncommon complication of FNA which, however, can be life threatening. *Case Presentation*. A 49-year-old lady presented with fever and neck pain 1 month after FNA with biochemical evidence of thyrotoxicosis. Repeat US of the thyroid showed interval enlargement of the thyroid nodule, and the culture of the cystic fluid of repeat FNA grew *Propionibacterium acnes*. She responded well to bedside aspiration and 2 weeks of antibiotic therapy without requiring surgical intervention. *Discussion*. Acute bacterial suppurative thyroiditis following FNA has been increasingly reported in immunocompetent hosts. There are 2 peculiar features in our case: a smoldering course caused by an indolent organism and a significant time lag between initial FNA and clinical presentation. On literature review, it was found that the onset of acute bacterial suppurative thyroiditis after FNA can range from a few days to up to 3 months. Clinicians should be aware of this complication even if FNA has been performed a few months ago. Thyroid US and US-guided FNA are useful initial investigations. Conventional management of acute bacterial suppurative thyroiditis has been surgery combined with antimicrobial therapy. However, recently, a more conservative approach has been reported to be effective in the treatment of acute bacterial suppurative thyroiditis as well.

**Conclusion:**

Proper infection control practices are necessary in performing the FNA. Initial management (conservative versus surgical) of acute bacterial thyroiditis should be based on the patient's clinical status and the extent of infective focus.

## 1. Introduction

Fine needle aspiration (FNA) of thyroid nodules is a simple, reliable, and inexpensive procedure performed on suspicious thyroid nodules found in thyroid ultrasound (US) [1]. We present an unusual complication of FNA causing acute bacterial thyroiditis. Our case is the second case of anaerobic acute bacterial thyroiditis following FNA reported in the available literature. This patient's clinical presentation is peculiar as she presented with thyrotoxicosis and had no known congenital piriform sinus fistula and immunodeficiency risk factors. We also reviewed the available literature on acute bacterial thyroiditis following FNA and outlined its pathophysiology and management.

## 2. Case Presentation

**Figure 1:**
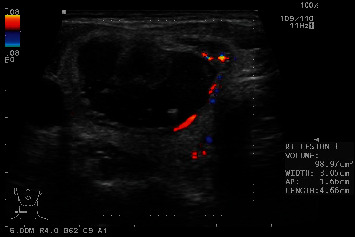
Ultrasound image before presentation showing the right mid to lower pole mixed cystic-solid thyroid nodule measuring 4.66 × 3.05 × 1.66 cm, with a well-defined margin and comet-tail artefact (consistent with the colloid nodule).

A 49-year-old female with the background history of hypertension, hyperlipidaemia, and presumed transient ischemic attack (TIA) presented to our endocrine clinic in November 2017 with an anterior neck lump of 1-year duration. The baseline thyroid function test was normal, and fine needle aspiration cytology (FNAC) of the thyroid nodule previously performed in another hospital revealed scanty yield of follicular cells consistent with nodular goitre. The initial US thyroid report and prior thyroid nodule dimension were unavailable. On examination, a right thyroid nodule was noted, measuring 3 cm by 4 cm. There was no palpably enlarged cervical lymph node, or tracheal deviation, and Pemberton's sign was negative. US thyroid performed in November 2017 showed a 4.7  cm × 3.1  cm × 1.7 cm mixed solid-cystic thyroid nodule in the right mid to lower pole, with comet-tail artefact and peripheral vascularity (Figure 1). In view of the size of the thyroid nodule and the lack of objective measure of growth (baseline nodule size not known), a repeat US-guided FNAC of this thyroid nodule was performed on 7 Feb 2018, and this showed a benign colloid nodule with cystic degeneration. As such, a follow-up plan at 1 year was made. The patient represented to our clinic on 6 March 2018, complaining of pain over the thyroid nodule and fever of 39 degree Celsius for 1-day duration. Upper respiratory tract infective symptoms were also present. Oral antibiotics (cloxacillin and clarithromycin) were started for the presumptive diagnosis of infected thyroid cyst. In view of the presence of pain from a patient with pre-existing thyroid cyst, the differential diagnosis was that of haemorrhage into the thyroid cyst. She was planned for close outpatient follow-up. However, the patient presented to the Emergency Department on 13 March 2018 for persistent fever and neck pain and was admitted for further treatment.

**Figure 2:**
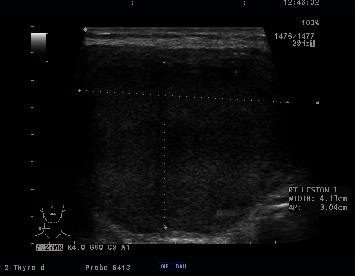
Ultrasound image at the time of presentation with neck pain and fever, showing the same thyroid nodule with increase in size measuring 4.95 × 4.13 × 3.04 cm and with interval development of isoechoic debris in the cystic fluid.

On admission, the patient had hand tremors and tenderness over her right thyroid nodule without any overlying skin changes or palpable cervical lymph nodes. She did not have any signs of airway compression. She was not in respiratory distress, had no tracheal deviation, and Pemberton's sign was negative. There were no features of thyroid eye disease. Vital signs were stable with a temperature of 36.6 degree Celsius, blood pressure of 140/77 mmHg, heart rate of 65/min, which was regular in rhythm, and oxygen saturation of 99% on room air. Investigations on admission showed a raised white cell count of 13.85 × 10^9^/L (reference range, 3.84–10.01 × 10^9^/L) with neutrophilia, raised ESR of 105 mm/hr (reference range, 3–9 mm/hr), raised fT4 of 25.7 pmol/L (reference range, 8–16 pmol/L), and suppressed TSH of 0.04 mIU/L (reference range, 0.45–4.5 mIU/L). TSH receptor antibody (TRAb), thyroid peroxidase antibody (anti-TPO Ab), and thyroglobulin antibody (anti-Tg) were negative. A thyroid uptake scan showed generalized reduced thyroid uptake suggestive of destructive thyroiditis and a large cold nodule in the right mid to lower pole of the thyroid gland. Repeat US thyroid revealed interval enlargement of the thyroid nodule which measured 5.0  cm × 4.1  cm × 3.0 cm with interval development of isoechoic debris in the cystic fluid (Figure 2). We were only able to aspirate 30 ml of fluid from the thyroid cyst under strict aseptic condition. Nonpurulent reddish-brown cystic thyroid fluid was sent for Gram stain and culture.

The patient was treated with intravenous Augmentin, nonsteroidal anti-inflammatory drugs (NSAIDs), and low-dose propranolol 20 mg two times a day with the presumptive diagnosis of infected thyroid cyst with thyrotoxicosis from destructive thyroiditis. Gram stain smear of the cystic fluid revealed numerous white blood cells and moderate Gram-positive rods. The cystic fluid culture grew *Propionibacterium acnes*. The patient remained afebrile and clinically stable. She completed 7 days of intravenous Augmentin after which she was discharged, and 7 day of oral amoxicillin was continued as outpatient treatment. At follow-up visit 1 week later, the patient was afebrile, clinically euthyroid, and her neck swelling was stable in size and no longer tender. The thyroid function test and inflammatory markers had normalized at follow-up review 3 weeks after discharge. She remained euthyroid and well without any recurrence of neck tenderness. Repeat US thyroid at 1 year showed markedly smaller right solid-cystic thyroid nodule, measuring 1.3 cm in the greatest dimension, and on US thyroid performed at 2 years, it remained stable in size.

## 3. Discussion and Literature Review

Our case represents a case of acute bacterial thyroiditis caused by *Propionibacterium acnes,* which featured two atypical clinical presentations: a smoldering course caused by an indolent organism and a significant time lag between initial FNA and clinical presentation.

We did a literature search on the PubMed database, as well as the Google search engine using the search terms, “FNA”, “thyroiditis,” and “abscess”. References were selected based on the identification of the relevance of the topic by the authors. The previous case reports of acute bacterial thyroiditis following FNA are presented in Table 1.

Acute bacterial suppurative thyroiditis is a rare disorder of the thyroid gland, representing only 0.1–0.7% of all thyroid disease [2]. Although infrequent, it can sometimes lead to serious life-threatening endocrine emergency, endangering the airway, causing cardiac arrhythmias and systemic sepsis, with or without thyrotoxicosis. Thyroid gland is relatively resistant to infections due to its rich vascularity, lymphatic supply, presence of complete fibrous capsule separating it from other nearby structures, high iodine, and hydrogen peroxide content. Infections of the thyroid gland usually occur in patients with underlying thyroid abnormalities such as multinodular goiter, thyroid neoplasms, autoimmune thyroiditis, or persistence of piriform sinus fistula (a tract extending from the apex of the piriform sinus in the hypopharynx to the thyroid gland, usually left-sided, and patients present at a younger age) or in immunocompromised patients such as those who are on immunosuppressant and those with HIV infection, diabetes, or inherited immunodeficiency [2, 3]. Most common organisms causing acute suppurative thyroiditis are Gram-positive aerobes (*Staphylococcus aureus* and *Streptococcus* sp.), followed by Gram-negative aerobes. These two groups constitute nearly 70% of all cases of acute suppurative thyroiditis. Anaerobes constitute only about 12% of all cases. Acute suppurative thyroiditis can rarely be caused by mycobacterial and fungal infections [3].

In earlier reviews, underlying thyroid abnormalities and immunocompromised status were two important risk factors for the development of acute suppurative thyroiditis [3, 4]. However, in a recent case series, spontaneous acute bacterial suppurative thyroiditis has been increasingly reported in immunocompetent patients without underlying structural abnormality such as pyriform sinus fistula [5]. Also, these patients had a more favorable prognosis than the earlier case series. This can be related to improved diagnostic ability in the recent years or under-reporting of acute bacterial thyroiditis in immunocompetent hosts in the previous years. In our patient, although recent FNA and the underlying thyroid nodule could be considered as risk factors for the development of acute bacterial thyroiditis, she did not have any immunodeficiency conditions. Therefore, acute bacterial suppurative thyroiditis following thyroid FNA should be considered as a rare but possible complication even in an immunocompetent host.

We reviewed our infection control practice after this incident. In our institution, thyroid FNA is performed only by experienced practitioners who run FNA clinic every week. The skin is cleaned with 70% alcohol, and we use a sterile disposable transducer probe cover. Although sterile gloves are not used, we ensure no-touch technique to the cleaned skin surface. Our technique is the same as that used in the United States and Europe [6, 7]. Our thyroid FNA service, which started in 2011, had performed an average of 236 thyroid nodule FNAs per year in the past 5 years, and this case is the first case of acute bacterial thyroiditis encountered. For the year of 2018, there were 247 thyroid nodule FNAs performed. As such, the rate of acute bacterial thyroiditis in our clinic in 2018 was 0.4%. No similar cases occurred after the index case.

In our patient, the acute bacterial thyroiditis is caused by *Propionibacterium acnes,* a slow-growing aero-tolerant anaerobic Gram-positive bacillus. It is a commensal of the human skin, gastrointestinal tract, conjunctiva, and external ear. Apart from its well-known link to pathogenesis of acnes vulgaris, it is now recognized as an opportunistic pathogen causing infection of prostheses, ventricular shunts, cardiovascular devices, the heart valves, and the eye [8]. Our case is the second case of acute bacterial thyroiditis due to *Propionibacterium acnes,* according to the available literature [9]. In both of the cases caused by *Propionibacterium acnes*, the presentation is more subtle compared to the typical presentation of acute bacterial thyroiditis. There was no spiking fever, overlying skin change, airway compressive symptoms, or sudden drastic increase in size of the thyroid nodules. All of these features can be contributed to the slow-growing nature and low pathogenic potential of *Propionibacterium acnes*. *Propionibacterium* species are susceptible to betalactam antibiotics, macrolides, lincosamides, tetracyclines, and fluoroquinolones. Penicillin *G* is the drug of choice. Resistant strains have developed to macrolides, lincosamides, and tetracyclines. [10].

Possible differential diagnoses in our patient include, but are not limited to, De Quervain's thyroiditis, hemorrhage into the thyroid cyst, acute suppurative thyroiditis, and aggressive thyroid cancer or thyroid lymphoma [11]. Release of thyroid hormones from damaged thyroid follicles can lead to thyrotoxicosis and reduced radioiodine uptake in destructive thyroiditis or acute suppurative thyroiditis. Neck tenderness or neck swelling progression and elevated circulating inflammatory markers can be present in all these conditions and cannot be used as discriminatory factors. Thyroid US and US-guided FNA are the most useful investigations in this clinical setting. Thyroid US features in acute bacterial thyroiditis may include heterogeneous echotexture of the thyroid gland and interval development of hypo-to hyperechoic debris in the thyroid cystic fluid. US-guided FNA will show blood in the case of hemorrhage, polymorphonuclear cell infiltration in the case of acute suppurative thyroiditis, multinucleated giant cells and mononuclear cell infiltration in the case of De Quervain's thyroiditis, and atypical or malignant cells in the case of malignancy. Gram stain smear and culture of FNA material will give additional information as in our patient. However, initial stabilization of the patient must always take precedence, and the role of other imaging such as neck CT scan to delineate the extent and complexity of abscesses or neck anatomy should be considered, especially when the size of the infective focus is large [2]. In the case of pediatric presentation, endoscopic hypopharyngoscopy, neck CT scan with trumpet maneuver, and barium swallow can identify piriform sinus fistula that predisposes to thyroid abscess development [2, 12, 13].

Another peculiar feature in our patient is the time lag of 1 month between the time of FNA and the time of presentation with fever and neck pain. In the available literature, the onset of acute bacterial suppurative thyroiditis after FNA can range from a few days to 3 months [14]. Prolonged subclinical course or subtle presentation was seen in cases of immunocompromised hosts or infection with a low virulent organism as in our case. The suspicion of acute suppurative thyroiditis in a patient with neck pain, swelling, and fever should always be borne in mind, especially if the patient has undergone FNA even a few months ago.

Conventional management of acute bacterial suppurative thyroiditis has been surgery combined with antimicrobial therapy [14]. Recently, a more conservative approach has been reported to be effective [9, 15, 16]. When we reviewed the case reports of acute bacterial suppurative thyroiditis following thyroid FNA, most used parenteral broad spectrum empirical antibiotic therapy with or without needle aspiration drainage as the initial management, similar to our case. Definitive surgery (open drainage or thyroidectomy) were required in a few cases, with main indications being impending airway obstruction in 2 cases [14, 17], underlying papillary thyroid cancer (PTC) in 1 case [18], and failed aspiration due to thick content of cyst in 1 case [19]. These scenarios underlined the importance of personalized management of acute bacterial suppurative thyroiditis based on clinical status of patients. Two case reports required surgery a few weeks after the initial presentation (catheter drainage in one case [20] due to recollection of pus and lobectomy in another case [21] due to persistent fever, leukocytosis, and elevated inflammatory markers). These cases demonstrate the importance of close follow-up in patients who are initially managed conservatively.

## 4. Conclusions

Acute bacterial suppurative thyroiditis following thyroid FNA is a rare but possible complication even in immunocompetent patients with no known risk factors. Hence, a proper infection control practice is necessary when performing the procedure. The presentation can be subtle and prolonged, sometimes mimicking subacute thyroiditis; it can also present as an emergency endangering the airway, with sepsis and/or thyrotoxicosis. Thyroid US and US-guided FNA are useful initial investigation modalities. Initial management (conservative versus surgical) should be based on the patient's clinical status and the extent of infective focus. Even in patients who are initially managed conservatively, the need for surgery should always be evaluated closely.

## Figures and Tables

**Table 1:** Case reports of acute bacterial thyroiditis following FNA in the literature.

Author	Published year	Causal organism	Risk factor	Thyroid function status at presentation	Time of FNA to presentation	Management
Isenberg [22]	1994	*Staphylococci*	Diabetes mellitus	Not stated	Not available	Not available
Wang et al. [14]	1997	*Escherichia coli*	On adjuvant chemotherapy for colon cancer and leukopenic	Euthyroid	3 months	Semiemergent thyroidectomy
Sun et al. [9]	2001	*Propionibacterium acnes*	No known risk factors	Not stated	Not stated	Pus aspiration 3 times and antibiotics for 28 days
Nishihara et al. [21]	2005	*Staphylococcus aureus*	Severe atopic dermatitis	Thyrotoxic	4 days	Antibiotics for 1 month followed by resection of the affected thyroid lobe due to persistent fever, raised inflammatory markers, and leukocytosis
Chen et al. [18]	2005	Sterile	Papillary thyroid cancer (PTC)	Euthyroid	Within a week	Parenteral antibiotics for 2 weeks followed by total thyroidectomy for PTC
Halenka et al. [17]	2008	*Escherichia coli*	No known risk factors	Euthyroid	3 days	Drainage of abscess with parenteral antibiotics followed by total thyroidectomy
Yildar et al. [20]	2014	Methicillin-sensitive *Staphylococcus aureus*	Diabetes mellitus	Thyrotoxic on the 7th day of treatment(Increased uptake in the thyroid gland surrounding the abscess in thyroid scintigraphy) etiology of hyperthyroidism was not discussed in the article	15 days	Needle aspiration of abscess 3 times and parenteral antibiotics for 14 days followed by oral antibiotics. Drainage with a 6F pigtail catheter on the 12th day after the end of parenteral antibiotics, due to further enlargement of the nodule. In view of sterile culture, no antibiotics were given at the time of catheter drainage
Unluturk U et al. [15]	2014	Sterile	No known risk factors	Not stated	3 days	Empirical antibiotics for 10 days
Tartaglia et al. [16]	2017	Not done	Psoriasis on methotrexate	Euthyroid	10 days	Parenteral empiric antibiotics (ceftazidime and teicoplanin) for 11 days followed by oral amoxicillin/clavlanic acid and levofloxacin for 10 days
Ar et al. [19]	2018	Staphylococci	No known risk factors	Thyrotoxic	3 weeks	Parenteral antibiotics, open drainage of the abscess, and excision of the cyst wall and right lobe of the thyroid on the 2nd day of admission due to failed aspiration and persistent fever in spite of antibiotic therapy
